# Supplementary figures and images for: Detecting the effect of genetic diversity on brain composition in an Alzheimer’s disease mouse model
Source: bioRxiv. 2023 Feb 28:2023.02.27.530226. Preprint. [Version 1] doi: 10.1101/2023.02.27.530226 (PMC10002670; doi:10.1101/2023.02.27.530226)

Supplemental Table 1

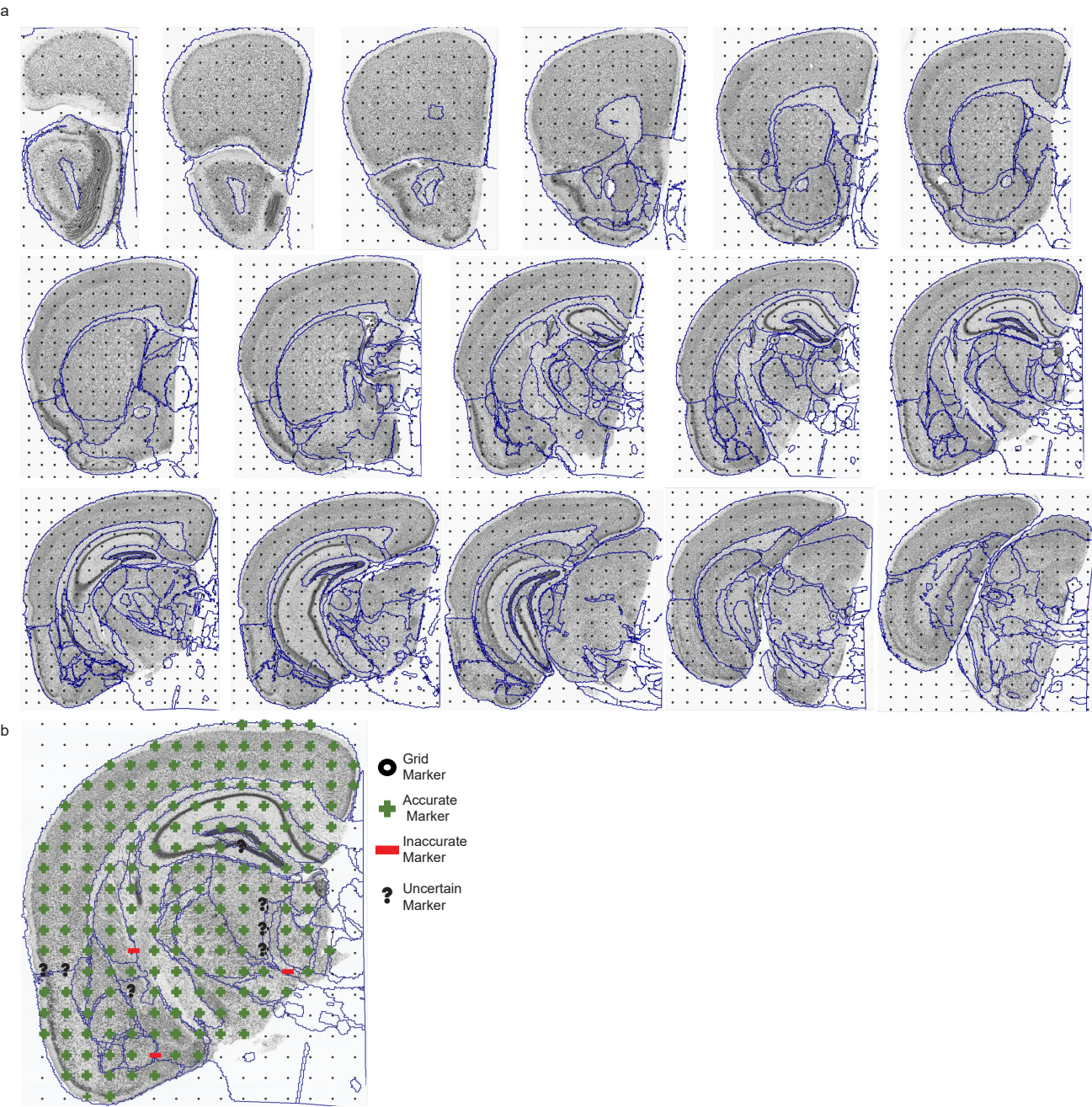

Supplemental Figure 2

a

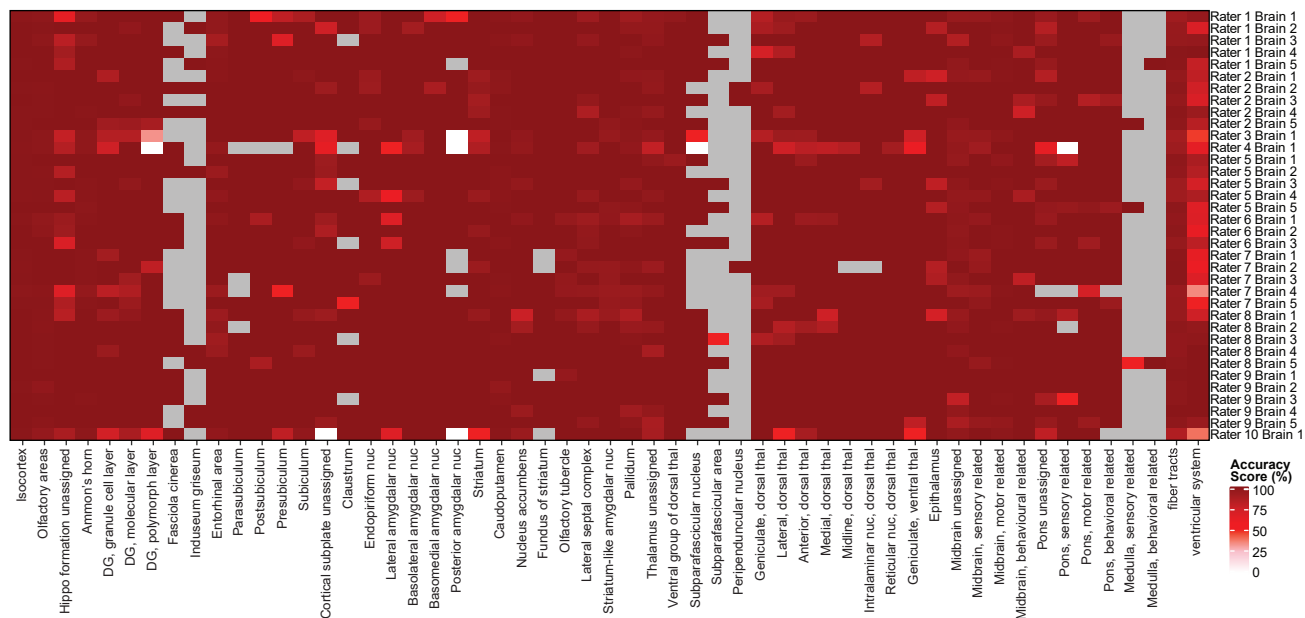

b.

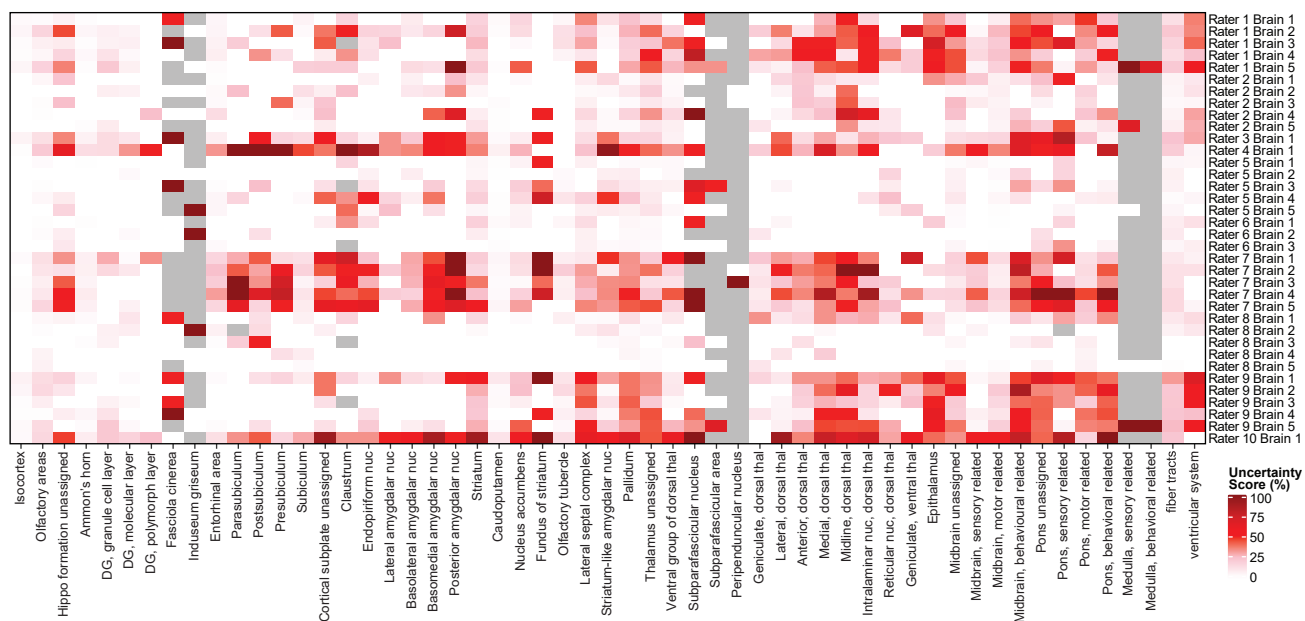

c

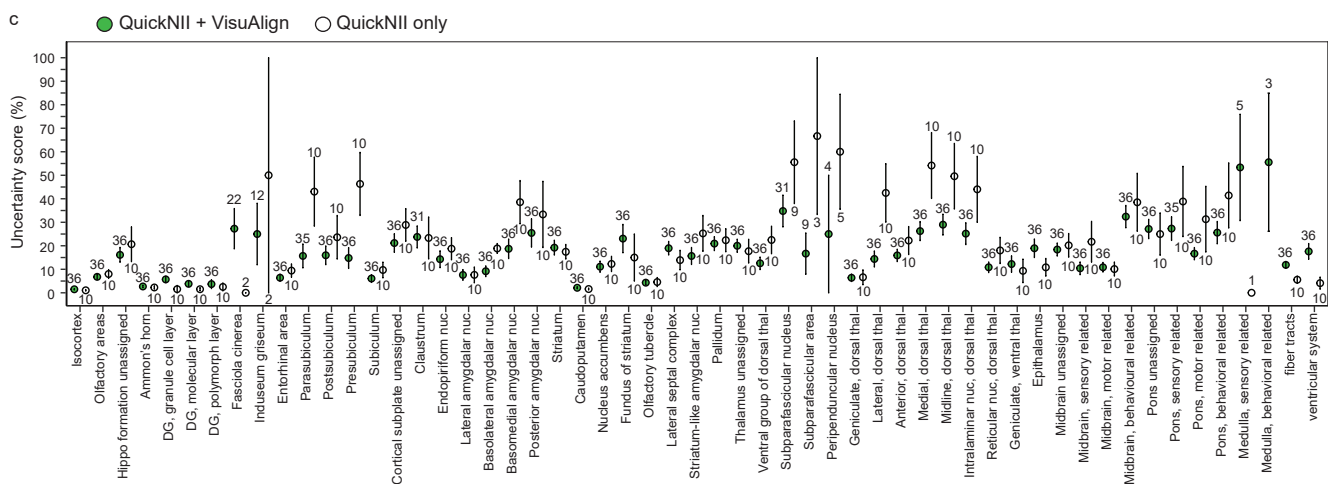

Supplemental Figure 3

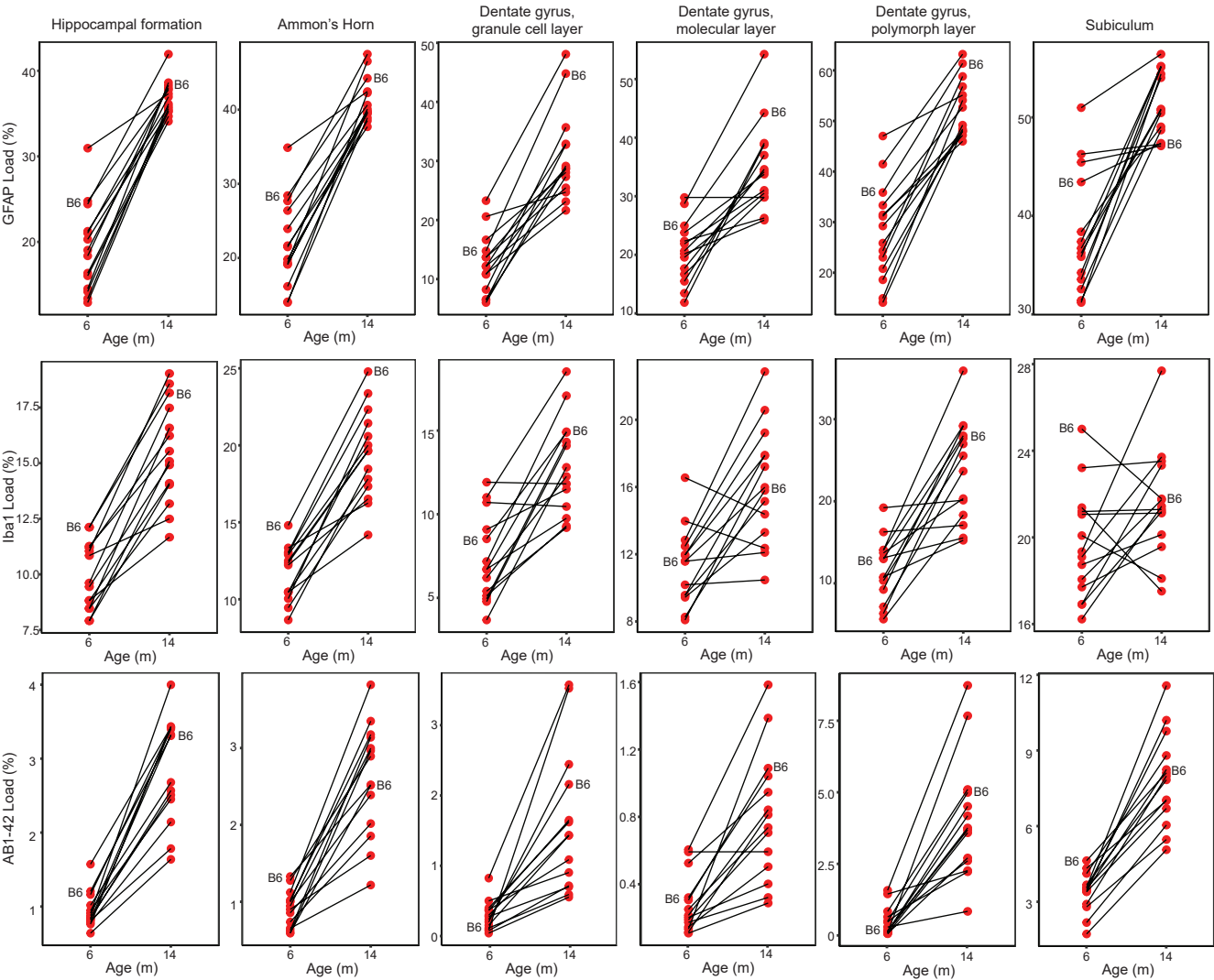

Supplement: Supplement 1 — Supplemental Figure 1: Intermediate hierarchy and QCAlign quality control assessment of atlas registration of thionine sections. a.) Intermediate hierarchy depiction over every thionine section of a representative brain following atlas registration using QuickNII and VisuAlign. Allen Mouse Brain Atlas CCFv3 regions were compiled to make an intermediate hierarchy that promotes the assessment of regional registration. b.) Representative quality control assessment of the atlas registration of a thionine slice in QCAlign. Raters assigned grid markers verifying the registration of each point as either accurate, inaccurate, or uncertain. Supplemental Figure 2: QCAlign scores achieved based on quality control assessment of intermediate hierarchy regions. a.) Heatmap of regional accuracy scores per rater per brain. b). Heatmap of regional uncertainty scores per rater per brain. Gray regions were not represented in the brain series and/or did not receive QCAlign scores for the measure. c.) Averaged uncertainty scores per intermediate hierarchy region after QuickNII registration alone (white) or after QuickNII and VisuAlign registration (green). Two raters scored the same 5 randomly selected brains after QuickNII registration alone, max n=10 per region (Raters: n= 2 per brain). Up to 10 raters scored the same 5 randomly selected brains after QuickNII and VisuAlign registration, max n=36 per region (Raters: n= 6–10 per brain). Dots represent the mean score across raters per region for 5 brains ±SEM, with the numbers labels representing the number of assessments contributing to each calculation (QuickNII alone labels are below white points, QuickNII + VisuAlign labels are above green points). Supplemental Figure 3. Variation in stain load exists among AD-BXD strains. Strain averages of a.) GFAP, b.) Iba1, and c.) AB1-42 load across the hippocampal formation and hippocampal intermediate hierarchy subregions. Points are mean load per strain. Each line connects a pair of strain av [file media-1.pdf]
